# Sensorimotor adaptation as a behavioural biomarker of early spinocerebellar ataxia type 6

**DOI:** 10.1038/s41598-017-02469-7

**Published:** 2017-05-24

**Authors:** Muriel T. N. Panouillères, Raed A. Joundi, Sonia Benitez-Rivero, Binith Cheeran, Christopher R. Butler, Andrea H. Németh, R. Chris Miall, Ned Jenkinson

**Affiliations:** 1 Nuffield Department of Clinical Neurosciences, University of Oxford, Oxford, United Kingdom; 2 Department of Experimental Psychology, University of Oxford, Oxford, United Kingdom; 3 Department of Physiology, Anatomy and Genetics, University of Oxford, Oxford, United Kingdom; 4 Department of Clinical Genetics, Churchill Hospital, Oxford University Hospitals NHS Trust, Oxford, United Kingdom; 5 School of Psychology, University of Birmingham, Birmingham, United Kingdom; 6 School of Sport, Exercise and Rehabilitation Sciences, University of Birmingham, Birmingham, United Kingdom

## Abstract

Early detection of the behavioural deficits of neurodegenerative diseases may help to describe the pathogenesis of such diseases and establish important biomarkers of disease progression. The aim of this study was to identify how sensorimotor adaptation of the upper limb, a cerebellar-dependent process restoring movement accuracy after introduction of a perturbation, is affected at the pre-clinical and clinical stages of spinocerebellar ataxia type 6 (SCA6), an inherited neurodegenerative disease. We demonstrate that initial adaptation to the perturbation was significantly impaired in the eighteen individuals with clinical motor symptoms but mostly preserved in the five pre-clinical individuals. Moreover, the amount of error reduction correlated with the clinical symptoms, with the most symptomatic patients adapting the least. Finally both pre-clinical and clinical individuals showed significantly reduced de-adaptation performance after the perturbation was removed in comparison to the control participants. Thus, in this large study of motor features in SCA6, we provide novel evidence for the existence of subclinical motor dysfunction at a pre-clinical stage of SCA6. Our findings show that testing sensorimotor de-adaptation could provide a potential predictor of future motor deficits in SCA6.

## Introduction

Spinocerebellar ataxia type 6 (SCA6) is a common form of autosomal dominant cerebellar ataxia presenting in adults^1^, corresponding to up to 31% of autosomal dominant ataxias in some countries^2^. It is caused by a pathological expansion of the polyglutamine-encoding CAG repeat in the *CACNA1A* gene on chromosome 19^3^ and it is 100% penetrant. The mutation primarily leads to the degeneration of Purkinje cells in the cerebellar cortex^4,5^, affecting first midline structures such as the vermis^6^. SCA6 is characterized by a late age of onset and a slow progression of symptoms including limb and gait ataxia, dysarthria and nystagmus^4^. In a recent review^7^, it was suggested that a certain threshold of degeneration is required before patients with spinocerebellar ataxias begin to experience symptoms. This view has been strengthened in a mouse-model study of SCA6, in which Purkinje cells loss occurs at 4 months without motor symptoms. In this model, motor dysfunctions only become apparent when the mice reach the age of 8 months^8^. The time lag between neuronal degeneration and symptom onset suggests that the initial cerebellar dysfunction is either functionally irrelevant or compensated for. An important alternative, however, is that functional motor changes on a subclinical level precede the onset of overt clinical motor manifestations.

The cerebellum is a key structure in sensorimotor adaptation, a critical process which progressively restores optimal motor performance when consistent errors are repeatedly encountered^9^. Indeed, brain imaging has shown the involvement of the cerebellum in various sensorimotor adaptation conditions^10–13^. Moreover, transiently modifying cerebellar function using non-invasive brain stimulation also affects sensorimotor adaptation^14–21^. Finally, numerous studies have demonstrated that individuals with cerebellar damage (lesion or degeneration) show impaired adaptation of limb and saccadic eye movements^22–36^. Given the key role of the cerebellum in sensorimotor adaptation, this process is an excellent candidate both to screen for pre-clinical deficits in SCA6 and to track disease progression in the clinical patients. The present study has two aims: (1) to establish whether changes in sensorimotor adaptation might be seen in pre-clinical SCA6 individuals and (2) to determine how the sensorimotor adaptation changes progress with the degeneration. Thus, sensorimotor adaptation could provide a novel clinical biomarker of SCA6 and a novel objective measure to track disease progression.

**Figure 1:**
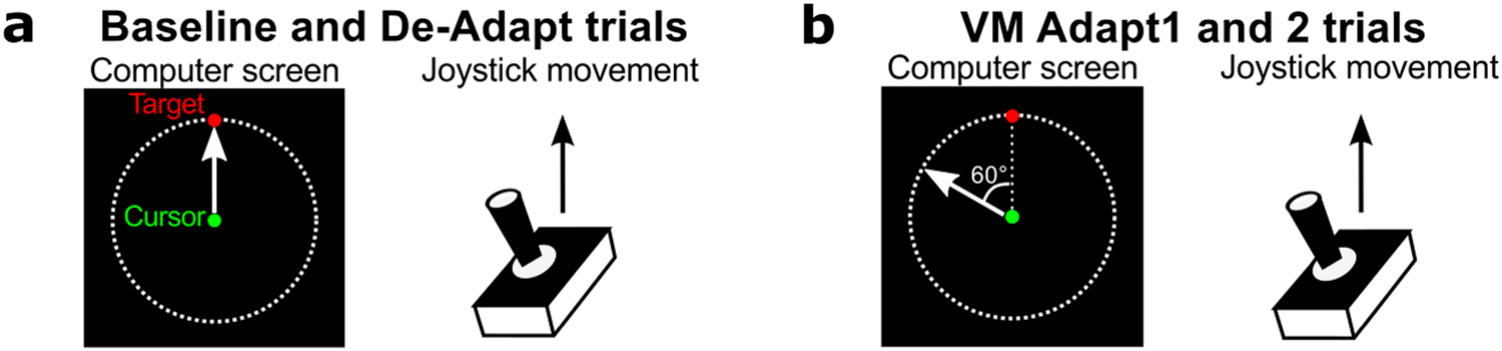
Relationship between the joystick movements and cursor movements for the different types of trials. (**a**) In the baseline and de-adaptation trials, the movement of the green cursor followed the exact path of the joystick movement. (**b**) For the visuomotor adaptation trials (VM Adapt1 and 2), the movement of the green cursor was rotated by 60 degrees anti-clockwise relative to the joystick movement. Note that the red target was presented randomly to one of 8 equidistant positions located on the dashed line circle.

Sensorimotor adaptation of the upper limb was tested using a version of the classic visuomotor rotation task^37–39^. In this task, participants must adapt their upper limb movement to a rotated visual feedback to successfully reach targets (Fig. 1b). Participants controlled a cursor on the computer screen using a joystick and made discrete 4.6 cm movements to targets at eight possible locations around the starting position. The visuomotor adaptation (VM Adapt) started after 50 trials, during which the direction of the cursor movement and of the joystick movement matched (Fig. 1a). This first phase allowed us to measure characteristics of the pre-clinical, clinical and control participants during normal movements. Participants then received two short sessions of adaptation (VM Adapt1 and VM Adapt2) separated by a 50-minute break. This allowed us to assess for adaptation performance and for the short-term benefit of a second adaptation session. Once adapted, the rotation was removed and individuals were submitted to a short de-adaptation phase assessing the after-effects of the adaptation. Adaptation abilities, short-term benefit of a second adaptation session and de-adaptation abilities were compared across all pre-clinical (PRE), clinical (CLIN) and control groups.

## Results

### Differences in demographics and clinical characteristics between PRE and CLIN individuals

As expected, the five patients in the PRE group were younger than the eighteen patients in the CLIN group (Table 1 – mean age: 43.6 ± 11.1 and 60 ± 6.1 years old respectively; Bootstrap independent samples t-test: t[5] = −3.56, p < 0.01). The ataxic symptoms, assessed using the International Cerebellar Ataxia Rate Scale (ICARS), were evident in the CLIN group, as indicated by higher total scores, while they were barely indicated in the PRE group (CLIN: 20 ± 10.3, PRE: 0.6 ± 0.5; t[17] = −7.99, p < 0.01). Gait and postural functions, quantified with the sub-score on the ICARS with the same name, were strongly affected in the CLIN group but again negligible in the PRE group (CLIN: 8.2 ± 5.1, PRE: 0.4 ± 0.5, t[18] = −6.36, p < 0.01). The kinetic functions in the CLIN patients were impaired while they were perfectly normal in the PRE individuals (6.5 ± 4.2 and 0.0 ± 0.0, respectively; t[17] = −6.60, p < 0.01). Of the twenty-three SCA6 individuals included in this study, sixteen patients were able to self-report the age where they noticed their first symptoms (Table 1), with a mean age of onset of 56 ± 6.3 years old.

**Table 1:** Demographic and clinical characteristics of the SCA6 individuals.

Patients	Sex	Age (years)	Self-reported Handedness	Disease Duration (years)	ICARS Total (/100)	ICARS posture and gait (/34)	ICARS kinetic (/52)	Gaze-evoked nystagmus	Saccadic dysmetria
PRE-1	F	58	Right	—	1	1	0	NO	NO
PRE-2	M	34	Right	—	1	0	0	Transient	NO
PRE-3	M	35	Left	—	0	0	0	NO	NO
PRE-4	F	53	Right	—	1	1	0	NO	NO
PRE-5	M	38	Right	—	0	0	0	NO	NO
CLIN-1	F	51	Right	—	8	6	1	NO	NO
CLIN-2	F	60	Right	13	12	3	2	Moderate	NO
CLIN -3	F	55	Right	1	16	11	3	Moderate	NO
CLIN -4	F	71	Right	2	27	14	6	NO	YES
CLIN -5	F	57	Right	6	21	6	9	NO	YES
CLIN -6	M	59	Right	6.5	38	13	16	Moderate	YES
CLIN -7	M	70	Right	6	30	13	9	Transient	YES
CLIN -8	M	57	Right	8.5	35	17	11	Moderate	NO
CLIN -9	M	65	Right	6	25	5	11	Transient	YES
CLIN -10	M	57	Right	8.5	16	3	7	Moderate	NO
CLIN -11	M	59	Left	7	11	2	4	Moderate	YES
CLIN -12	F	74	Right	11	39	17	12	Moderate	YES
CLIN -13	M	68	Right	—	9	4	4	Transient	NO
CLIN -14	M	60	Left	2	18	9	4	Transient	YES
CLIN -15	F	61	Right	10	20	10	5	Transient	NO
CLIN -16	M	61	Right	5.5	14	10	4	NO	NO
CLIN -17	M	65	Right	7	15	3	8	NO	NO
CLIN-18	M	66	Left	4.5	6	2	1	Transient	NO

PRE: pre-clinical individuals; CLIN: clinical patients; ICARS: International Cerebellar Ataxia Rate Scale. Gaze-evoked nystagmus (GEN) and saccadic dysmetria were assessed with the ICARS. The scale assesses whether the GEN was present, transient, moderate or severe. It also allows reporting the presence or absence of saccadic dysmetria. Disease duration was self-reported as the time where the first symptoms were noticed by the patient.

### Movement performance in baseline

**Figure 2:**
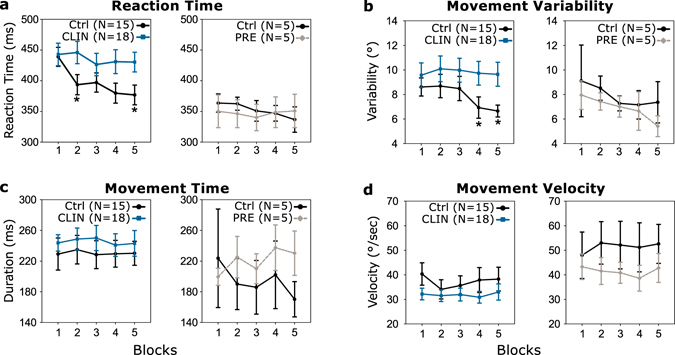
Movement kinematics in baseline. Movement reaction time (**a**), variability (**b**), time (**c**) and velocity (**d**) are shown for the 5 baseline blocks (average across 10 trials) for the CLIN patients (blue squares and lines), the PRE participants (grey diamonds and dashed lines) and their respective age-matched controls (Ctrl: black disks and lines). Error bars are standard errors of the mean. Statistical differences are represented as follows: *p < 0.05 (Significant ANOVAs followed by Bootstrap independent samples t-test).

**Figure 3:**
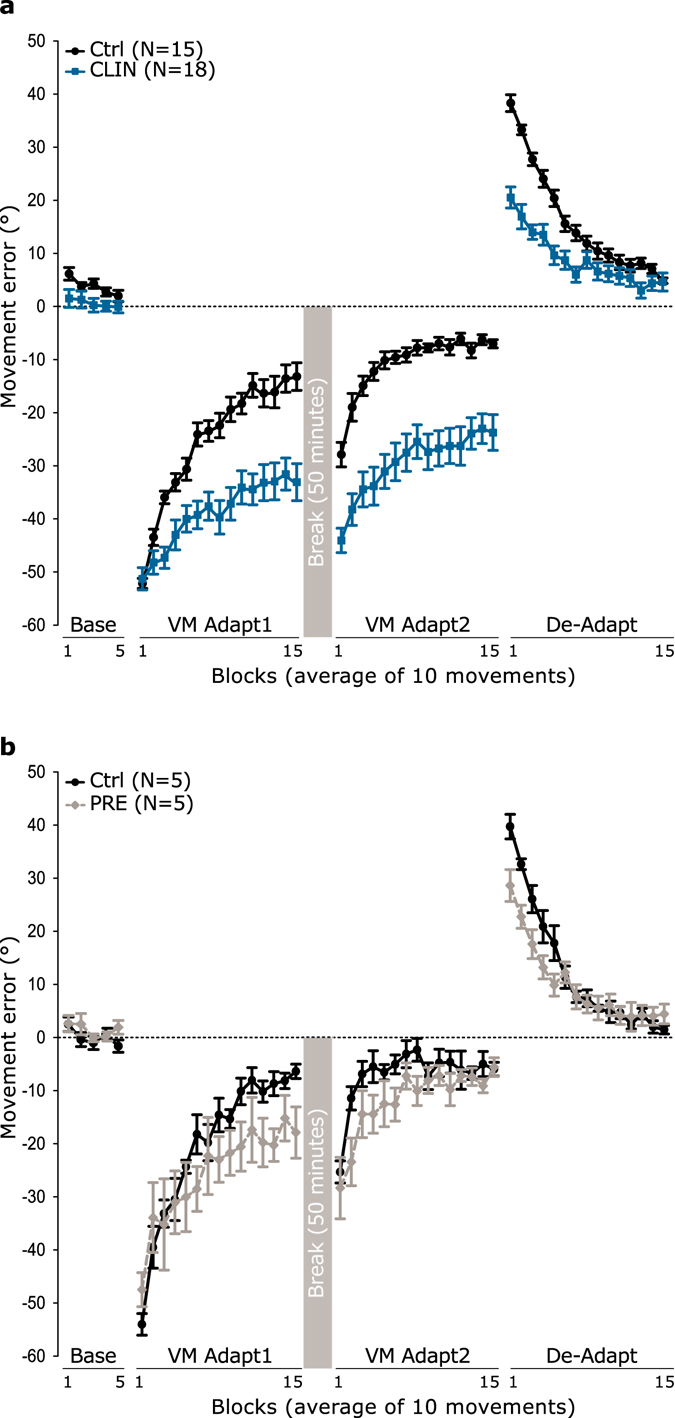
Movement error in baseline, adaptation and de-adaptation phases for the CLIN patients (**a**) and the PRE individuals (**b**). Movement error in degrees is represented in blocks of 10 trials throughout the different experimental phases: baseline, visuomotor adaptation 1 and 2 (VM Adapt1 and VM Adapt2) and de-adaptation (De-Adapt) for the CLIN patients (blue squares and lines), the PRE individuals (grey diamonds and dashed lines) and their respective control groups (Ctrl: black disks and lines). The 50-minutes break is represented by a grey square between VM Adapt1 and VM Adapt2. Negative (positive) errors correspond to anti-clockwise (clockwise) deviation relative to the target. Error bars are standard errors of the mean.

Because the PRE and CLIN groups were different in term of age, their performances were compared to two different age-matched controls groups. Before starting the adaptation phase, all subjects performed 50 baseline trials (subsequently divided into 5 blocks of 10 trials for analysis) during which there was no rotation between the cursor and joystick movements. The PRE individuals did not differ from their age-matched controls group in term of reaction time (Fig. 2a), movement variability (Fig. 2b), movement time (Fig. 2c), velocity (Fig. 2d) and movement error (Fig. 3 – two-way mixed design ANOVAs, no Group or Group × Block interaction: F[4,32] < 1.83, p > 0.15). However, the baseline trials revealed some differences between the movement kinematics of controls and CLIN patients in the unperturbed environment. The reaction times of the CLIN patients were longer for blocks 2 and 5 compared to those of the controls (Fig. 2a – Group effect: F[1,31] = 3.02, p = 0.09; Block effect: F[4,124] = 5.92, p < 0.001; Group × Block interaction: F[4,124] = 3.00, p < 0.05; Bootstrap independent t-tests: p < 0.05). Movement variability, measured as the standard-deviation of movement error across blocks of 10 trials, was significantly larger in CLIN patients than in the control participants (Fig. 2b – Group effect: F[1,31] = 9.40, p < 0.05). The difference in variability between the CLIN group and control group was present throughout the entire baseline but became significant only toward the end of this phase (blocks 4 and 5: Bootstrap independent t-tests: p = 0.05 and p < 0.05 respectively). Movement velocity and movement time was similar between the CLIN patients and their control group (Fig. 2c and d – Group effect: F[1,31] < 1.25, p > 0.27 and Group × Block interaction: F[4,124] < 1.89, p > 0.12). Movement errors slightly decreased in both groups with practise across the baseline trials (Fig. 3 – Block effect: F[4,124] = 3.49, p < 0.01). Movement error was slightly higher in the control group than in the CLIN group for the blocks 1, 3 and 4 of the baseline (Group effect: F[1,31] = 5.66, p < 0.05; Group × Block interaction: F[4,124] < 1, p > 0.50; Bootstrap independent t-tests: p < 0.05). Note however that by the end of the baseline trials, both groups performed the task similarly (block 5: Bootstrap independent t-test: t[31] = 1.35, p > 0.18).

Thus, PRE individuals performed in baseline similarly to age-matched controls, while patients with clinical SCA6 had higher variability and longer reaction times in the baseline condition as compared to the baseline performance of control participants. Movement accuracy in the baseline condition differed slightly between CLIN patients and age-matched controls at the beginning of the task, but this difference was lost by the end of baseline.

### Error reduction in VM Adapt1 phase was impaired for the CLIN group

Immediately after baseline, participants were exposed to a 60° anti-clockwise rotation of the cursor movement relative to the joystick movement, for the 150 trials of the VM Adapt1 phase. When this visuomotor perturbation was introduced, all groups produced large movement errors similar in magnitude to the rotation and these errors progressively decreased during VM Adapt1 to different extend depending on the group (Fig. 3). These variations in error reduction across group were evaluated by submitting the movement errors in this phase to two separate mixed-design ANOVAs with the between-subject factor Group (Control, SCA individuals) and the within-subject factor Block (1 to 15). The CLIN patients were only able to partially compensate for the perturbation during VM Adapt1, while the age-matched controls drastically decreased their error (Fig. 3a – Block effect: F[5,155] = 63.92, p < 0.001; Group effect: F[1,31] = 20.57, p < 0.001; Group × Block interaction: F[5,155] = 6.93, p < 0.05). Indeed, the mean error of the CLIN group was only reduced from −51° to −33° (paired samples t-test test comparing error between blocks 1 and 15: t[17] = −5.27, p < 0.001) while it was decreased from −52° to −13° in the age-matched control group (paired samples t-test: t[14] = −18.20, p < 0.001). The difference in error between the CLIN group and control group reached statistical significance by the third block of VM Adapt1 (Bootstrap independent t-tests: p < 0.05).

The movement errors of the PRE individuals and of their age-matched controls progressively diminished during VM Adapt1 (Fig. 3b – Block effect: F[3,27] = 37.44, p < 0.001). This reduction appeared to be quite similar between the two groups (Group effect: F[1,8] = 1.27, p = 0.29; Group × Block interaction: F[3,27] = 2.54, p = 0.07). Note that there is a trend for the PRE group to start reducing their error as quickly as the control group but to reach the end of VM Adapt1 with a larger residual error than the controls. Indeed, the mean error of the PRE group was reduced from −48° to −18° (related-samples Wilcoxon signed rank test comparing block 1 and 15, p < 0.05) while it was decreased from −54° to −6° in the age-matched control group (related-samples Wilcoxon signed rank test comparing block 1 and 15, p < 0.05). The difference in performance between PRE individuals and age-matched controls was only significance for block 13 of VM Adapt1 (Bootstrap independent samples t-tests, p < 0.05).

In summary, patients in the CLIN group showed a strong deficit in the adaptation to the visuomotor rotation, whereas the PRE individuals tended to start the adaptation phase well, but do not reach the same level of adaptation as the age-matched controls, progressively falling behind as the adaptation block continues.

### Correlation between adaptation performance in VM Adapt1 and ataxic symptoms

**Figure 4:**
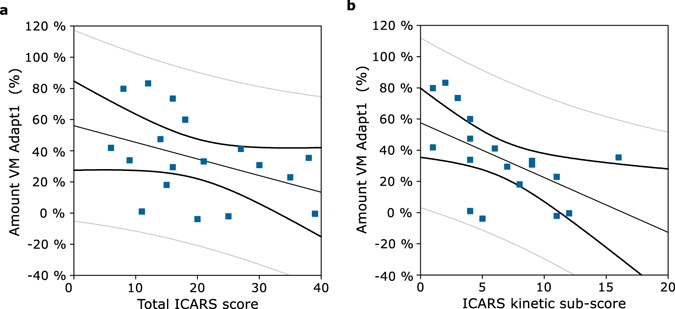
Correlations between the amount of adaptation in VM Adapt1 and patient symptoms. The amount of adaptation reached at the end of the first adaptation phase (VM Adapt1), calculated in percent, was correlated with the CLIN patients’ total ICARS scores (**a**) and with the CLIN patients’ ICARS kinetic sub-scores (**b**). Correlations are represented with the confidence (black thick lines) and prediction (grey thin lines) intervals.

To evaluate the relationship between disease severity and degree of adaptation, we correlated the percentage of adaptation in VM Adapt1 to the total ICARS score of the CLIN patients. There was a significant negative correlation between the patient’s overall symptoms and their level of adaptation in VM Adapt1 (Fig. 4a – one-tailed Pearson correlation: r[18] = −0.40, p < 0.05). A similar significant negative correlation was found between the kinetic sub-score of the ICARS and the level of adaptation reached at the end of VM Adapt1 (Fig. 4b – one-tailed Pearson correlation: r[18] = **−**0.54, p < 0.01). Thus, the most symptomatic patients, as measured by the ICARS score, have the most impaired adaptation.

### Error reduction in VM Adapt2 phase is also impaired in the CLIN group

After a resting break of 50 minutes, participants performed a second adaptation phase (Fig. 3 – VM Adapt2) identical to the first one (60° counter-clockwise rotation between cursor and joystick movements, 150 trials). We first compared the error across SCA6 individuals and their age-matched controls between the first block of VM Adapt1 and the first block of VM Adapt2. Movement error was significantly smaller at the beginning of VM Adapt2 relative to VM Adapt1 for all four groups (Phase effect: F[1,8] > 63.42, p < 0.001), showing that some of the adaptation was retained during the 50 minutes break. On one hand, PRE individuals and their control group did not differ from each other at the beginning of the two adaptation phases (Group effect: F[1,8] < 1, p = 0.68; Group × Phase interaction: F[1,8] = 2.52, p = 0.15). On the other hand, patients in the CLIN group started VM Adapt2 with a significantly larger error than their control group (Group effect: F[1,31] = 9.53, p < 0.01; and Group × Phase interaction: F[1,31] = 31.95, p < 0.001). This difference of error at the start of this second adaptation phase relates to the strongly reduced adaptation during the VM Adapt1 phase for the CLIN patients.

Next, we investigated the error reduction during the VM Adapt2 phase separately for the CLIN and PRE groups. As in VM Adapt1 (Fig. 3a), movement error decreased during VM Adapt2 in both CLIN group and their age-matched control group (Block effect: F[7,207] = 44.35, p < 0.001). However, movement error was significantly larger for the CLIN patients than for the control group throughout the entire phase (Group effect: F[1,31] = 29.85, p < 0.001, Bootstrap independent samples t-tests: p < 0.01). Indeed, during VM Adapt2, error significantly decreased in the control group from −28° to −7° (paired samples t-test comparing the errors between block 1 and 15: t[14] = −11.57, p < 0.001) and the error was also significantly reduced in the CLIN group but only from −44° to −24° (t[15] = −7.58, p < 0.001). In both CLIN and control groups, movement error in the last block of VM Adapt2 was significantly less than the movement error reached in the last block of VM Adapt1 (ANOVA comparing the last block of VM Adapt1 and of VM Adapt2 – Phase effect: F[1,31] = 28.05, p < 0.001), showing a cumulative effect of the two adaptation sessions in both groups.

Despite a non-significant tendency to adapt less at the beginning of the VM Adapt2 phase, the PRE group was able to reduce their error by the end of this phase to a level similar to their age-matched control group (Fig. 3b – Block effect: F[4,32] = 17.94, p < 0.001; Group effect: F[1,8] = 2.47, p = 0.15; Block × Group interaction: F[4,32] = 1.78, p = 0.16). Indeed, the error significantly decreased from −25° to −6° in the control group and from −28° to −6° in the PRE group (related-samples Wilcoxon signed rank test comparing block 1 and 15, p < 0.05). Note that when comparing the performance at the end of VM Adapt1 and 2 between PRE and control groups, there was only a cumulative effect of the two adaptation sessions in the PRE but not the control group (Phase interaction: F[1,8] = 11.39, p < 0.01; Group × Phase interaction: F[1,8] = 9.99, p < 0.05). These results suggest that the tendency for a slower adaptation in VM Adapt1 of the PRE individuals normalised throughout this second adaptation phase.

In summary, patients in the CLIN group adapted less in VM Adapt2 than their age-matched controls, as they had in VM Adapt1. On the contrary, individuals in the PRE group adapted similarly to control participants. Both PRE and CLIN groups benefited from this second adaptation session, as their performance was improved by the end of VM Adapt2 compared to the end of VM Adapt1.

### De-Adaptation performance is reduced in both PRE individuals and CLIN patients

The experiment ended with the de-adaptation phase (Fig. 3 – De-Adapt). In this phase, the cursor rotation was removed and movement of the joystick once again matched the movement of the cursor. Having adapted to the rotation during VM Adapt1 and 2, participants now produced errors in the opposite direction to those seen in the adaptation phase, representing after-effects from the previously adapted cursor rotation. The positive error progressively decreased for all four groups of participants during De-Adapt (Block effect: PRE ANOVA: F[4,29] = 65.62, p < 0.001; CLIN ANOVA: F[6,182] = 111.64, p < 0.001) and converged toward a final error of ~4°. However, there was a large difference between the amount of error seen at the start of this phase between the SCA6 participants and the control groups. As expected the CLIN patients (Fig. 3a), who adapted little in VM Adapt1 and 2, started the De-Adapt phase with smaller errors compared to the age-matched controls, which in turn led to the two groups returning to baseline performance at different times during the De-Adapt phase (Group effect: F[1,31] = 18.30, p < 0.001; Group × Block interaction: F[6,181] = 14.25, p < 0.001). Interestingly, the PRE patients (Fig. 3b) who reached the same level of adaptation as the control group at the end of VM Adapt2 also started the De-Adapt phase with smaller errors than the control (Group effect: F[1,8] = 2.44, p = 0.16; Group × Block interaction: F[4,29] = 4.10, p < 0.05). The difference between PRE and control was significant for the first two blocks of De-Adapt (Bootstrap independent samples t-test: p < 0.05) and close to be significant for the following 3 (p = [0.07–0.09]).

In summary, the clinical SCA6 patients who adapted poorly had smaller after-effects than their age-matched controls. However, despite reaching the same adaptation level as their age-matched controls, the pre-clinical individuals also presented with reduced after-effects and de-adaptation performance.

### Adaptation and De-Adaptation indices

**Figure 5:**
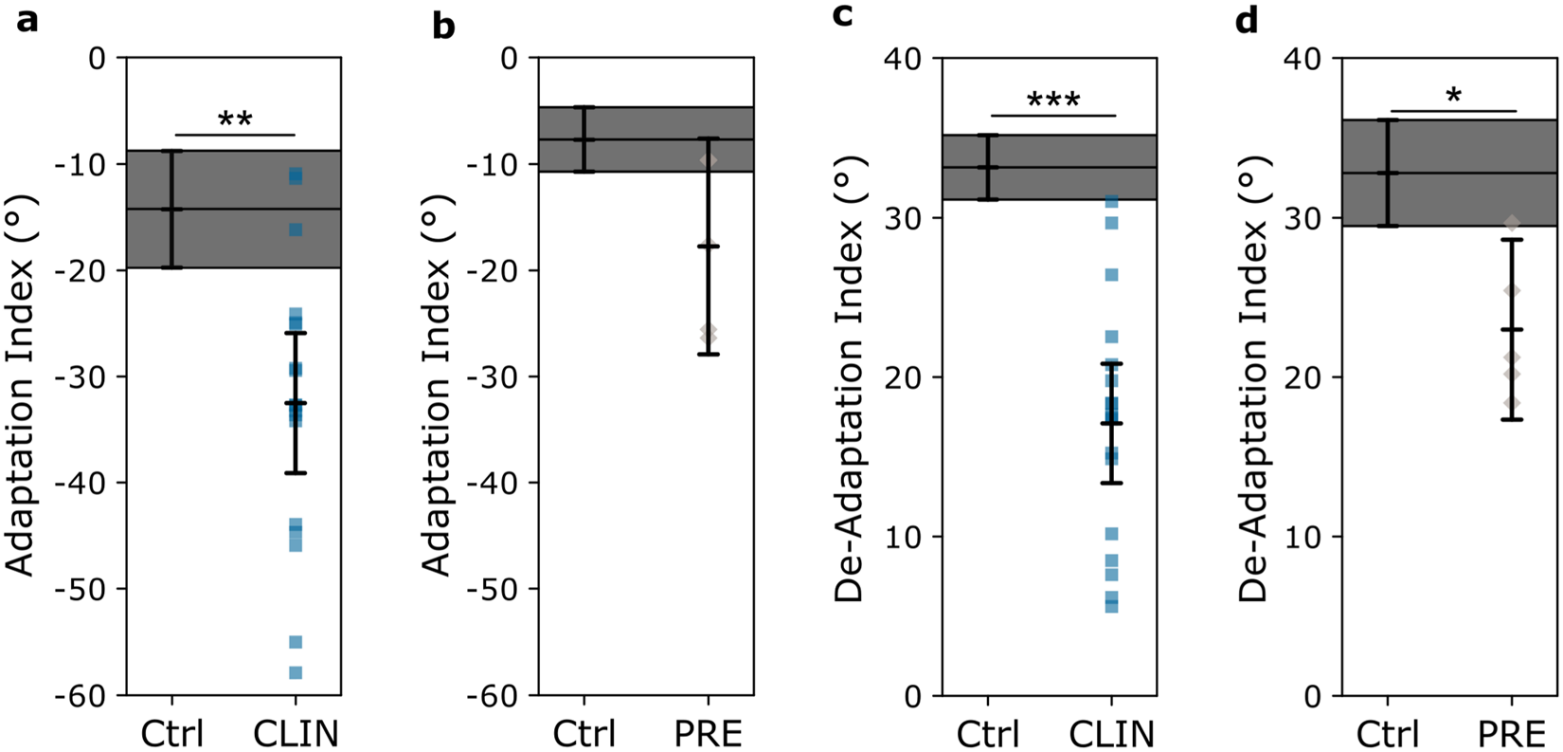
Adaptation Index (AI) and De-Adaptation index (DeAI) for the CLIN patients (**a,c**) and the PRE individuals (**b,d**). The individual values of the AI and DeAI are represented for the CLIN patients (blue squares) and for the PRE individuals (grey diamonds). Error bars represent the 95% confidence interval of the mean. The grey shaded area corresponds to the 95% confidence interval of the mean for the control groups. Statistically significant differences between control groups and patients are represented as follows: *p < 0.05, **p < 0.01 and ***p < 0.001 (Bootstrap independent samples t-test).

To capture the observed differences in performance in VM Adapt1 and De-Adapt between the individuals with SCA6 and their respective control groups, we calculated Adaptation and De-Adaptation Indices (AI and DeAI, respectively) by averaging movement error respectively across the last 30 trials of VM Adapt1 and the first 30 trials of De-Adapt for each participant (Fig. 5). For the adaptation phase, the CLIN group had a smaller index than their age-matched control group (Fig. 5a – Bootstrap independent samples t-test: t[25] = 4.39, p < 0.01) while the AI of the PRE individuals was similar to the one of their control group (Fig. 5b – Bootstrap independent samples t-test: t[5] = 2.64, p = 0.12). Note that 3 CLIN patients and 2 PRE individuals had performance that was inside the 95% confidence interval of their respective control groups. These results show that the adaptation of the CLIN patients was consistently reduced relative to the one of the control, on an individual basis, while the adaptation level of the PRE patients was not a strong predictor of their clinical status.

During the de-adaptation phase, the DeAI of the CLIN group was significantly reduced relative to their age-matched control group (Fig. 5c – Bootstrap independent samples t-test: t[31] = 7.99, p < 0.001). However, this did not correlate with the ataxic symptoms (one-tailed Pearson’s correlation: |r[18]| < 0.21, p > 0.20). Similarly, the DeAI of the PRE group was significantly smaller than the one of their control group (Fig. 5d – Bootstrap independent samples t-test: t[6] = 4.16, p < 0.05). If we normalise the DeAI by the amount of learning achieved at the end of the second session of adaptation, we again find that this index is significantly reduced for both CLIN and PRE individuals relative to the controls (Bootstrap independent samples t-test: t[31] = 3.67, p < 0.001 and t[8] = 3.69, p < 0.05 respectively). The DeAI of all 18 the CLIN patients, and of four of the five the PRE individuals was outside the 95% confidence interval of their control group mean (Fig. 5) demonstrating that this index could consistently differentiate individuals with SCA6 from the control participants.

### Saccadic deficits do not affect visuomotor performance in CLIN patients

To perform the visuomotor task, participants had to made saccadic eye movements between the target central and peripheral positions (sub-tending a visual angle of ~3.7°). Oculomotor disorders such as nystagmus (involuntary eye movements) and saccadic dysmetria could then lead to enhanced difficulties in realising the visuomotor task^40^. We therefore report the eye movements deficits of the patients, as assessed with the ICARS oculomotor dysfunction sub-score (Table 1). None of the patients had nystagmus when looking straight ahead (primary position nystagmus). One PRE individual and thirteen CLIN patients presented with gaze-evoked nystagmus (GEN). Zero and eight individuals in the PRE and CLIN groups respectively had some levels of saccadic dysmetria.

Patients’ oculomotor dysfunctions could have altered the processing of visual information about the target and/or the cursor position and could then partially account for the CLIN patients’ impaired performance. Because of the small visual eccentricity of the target (~3.7°), it is unlikely that the participants experienced gaze-evoked nystagmus. However, saccadic dysmetria may have interfered with the visual acquisition of the target and/or the cursor. To evaluate whether this was the case, we compared the performance of the 8 CLIN patients who presented saccadic dysmetria with the 10 patients who did not. We then performed three separate ANOVAs on the movement errors in VM Adapt1, VM Adapt2 and De-Adapt with the within-subject factor Block (1, …, 15) and the between-subjects factor Group (CLIN with NO Saccadic dysmetria vs CLIN with Saccadic dysmetria). For all three phases of the experiment, the performance of the CLIN patients was similar whether they had saccadic deficits or not (Group effect: F[1,16] < 1, p > 0.33; Group × Block interaction: F[5,76] < 1.40, p > 0.24). This shows that the performance of the visuomotor adaptation task was independent of the presence of saccadic impairments.

## Discussion

In this study, we demonstrate that SCA6 patients with clinical symptoms have a limited capability to adapt their upper limb movements in response to a large visuomotor rotation. Our results and those from previous studies (see Introduction) provide strong evidence that although cerebellar damage does not completely abolish the ability to adapt visuomotor behaviour, it dramatically reduces it. We have also revealed that this reduced sensorimotor adaptive ability correlates negatively with clinical ataxic symptoms. These results imply that the most symptomatic SCA6 patients are also the most impaired with respect to sensorimotor adaptation. The correlations in this study concur with those on ataxic patients of mixed aetiology^35,41^ and suggest that, from a clinical perspective, adaptive abilities could be a useful measure to track disease progression in SCA6.

When we consider the reduced ability to adapt in the clinical SCA6 patients, we should acknowledge that oculomotor deficits could, at least partially, be responsible for the deficits in the visuomotor rotation task. Indeed, difficulties in visually acquiring the target or the cursor due to saccadic dysmetria or gaze-evoked nystagmus (GEN) could impact the performance of the upper limb movements^40^. However, three arguments allow us to dismiss this possibility. Firstly, the accurate movements of the SCA6 individuals in baseline already suggest that oculomotor deficits do not affect their upper limb movement performance. Secondly, the target eccentricities in our task were very small (~3.7°) and GEN is usually evoked at eccentricities of 20° or more^42^. Thirdly, the equivalent level of adaptive performance of the SCA6 patients with known saccadic dysmetria and those without suggest that adaptive performance was not influenced by oculomotor dysfunctions. Thus, the visuomotor adaptation deficits of the SCA6 patients are most likely a direct result of cerebellar pathology and are not a by-product of cerebellar-dependent oculomotor deficits.

Unlike the SCA6 patients with manifest clinical symptoms, the ability of the pre-clinical carriers to adapt is partially reduced but still quite similar to that of controls. Indeed, despite the fact that the PRE individuals tended to adapt less during VM Adapt1, they were still able to reduce their error to the same extent as their control group by the end of the second phase of adaptation. However, these same pre-clinical participants (along with the clinical SCA6 patients) display significantly reduced de-adaptation performance compared to controls. To better capture the differences in de-adaptation performances across the clinical and pre-clinical SCA6 individuals and their respective control groups, we calculated a De-Adaptation Index (DeAI). By averaging the movement error across the first 30 trials of the de-adaptation phase we are capturing two aspects of this phase: the after-effects of the adaptation, which is the amount of error at the very beginning of the De-adaptation phase, and the ability to de-adapt from this newly acquired visuomotor remapping. Because the SCA6 individuals with clear symptoms adapted slower than their age-matched controls, they would be expected to retain less adaptation and so have reduced after-effects. Moreover, because their adaptation is slower, they may also de-adapt slower than their controls. Thus, the reduced DeAI in the CLIN individuals may be explained by a main deficit in adaptation, leading to reduced after-effects. However, the PRE group show equivalent adaptation to the controls by the end of the second adaptation phase, and yet still show reduced de-adaptation performances. One potential explanation for this reduced de-adaptation could be that because the PRE individuals reached the same adapted level than the control later in VM Adapt2, the memory of this newly acquired visuomotor rotation may not have had enough trials to be strongly consolidated. Therefore, when the perturbation is removed, the after-effects could washout more quickly, resulting in a smaller DeAI in this group. Using this novel measure, we show that not only did pre-clinical individuals have a significantly smaller mean index than the control participants at the group level, but the individual values of the DeAI for all pre-clinical individuals, except one, were below the 95% confidence interval of the control participants. Therefore, in this cohort of patients, reduced after-effect can be used to separate pre-clinical SCA6 individuals with cerebellar dysfunctions from non-SCA6 control participants, even though these individuals display no or minimum overt clinical symptoms. Three previous studies^43–45^ have reported heterogeneous oculomotor dysfunctions in pre-clinical SCA6 individuals and/or some level of gait deficits at the group level. On the contrary, our quantitative behavioural approach was able to objectively detect early and subtle deficits in upper limb sensorimotor performance in the pre-clinical SCA6 individuals, not otherwise identified using current subjective clinical scale. It should be noted that only one of the five pre-clinical individuals presented with ocular deficits, and so the DeAI could then be more sensitive in differentiating healthy individuals from pre-clinical SCA6 than oculomotor examination. Moreover, as the DeAI was reduced in both PRE and CLIN individuals and did not correlate with the ataxic symptoms of the CLIN patients, this index may be an indicator of the presence of the overall cerebellar dysfunction rather than an indicator of the disease progression.

SCA6 results from the slow degeneration of Purkinje cells of the cerebellar cortex^4,5^. In a mouse model, it has been shown that Purkinje cell loss precedes apparent outward motor symptoms by 4 months^8^. Our results, in one of the largest cohort of SCA6 so far reported, suggest that neuropathological changes in the human cerebellum may give rise to subtle upper limb sensorimotor deficits well before motor symptoms become clinically manifest. It is interesting to speculate that these deficits may be the result of early primary cerebellar pathology outside of the vermis. Indeed, evidence from imaging and patient studies have implicated the superior part of the cerebellar hemispheres, including lobule IV, V and Crus I, with visuomotor adaptation abilities of the upper limb^46–48^. Furthermore, the lateral superior lobules V and VI were shown to be involved in the after-effect of sensorimotor adaptation^48^. Despite evidence that degeneration in SCA6 first manifests in the vermis^4,6,49^, our results raise the possibility of a more diffuse pre-clinical process also affecting the lateral superior cerebellum, including lobules V and VI, leading to a tendency for diminished adaptation and resulting in strongly impaired after-effects. As the disease progresses, the degeneration may extend to more superior and inferior parts of the cerebellum, such as the lateral lobule IV and Crus I, to a point beyond functional compensation and then result in dramatic deficits in error reduction during adaptation.

To conclude, in this large study of motor features in SCA6, we provide novel evidence for the existence of subclinical motor dysfunction at an early pre-clinical stage of SCA6 by demonstrating significantly reduced de-adaptation performance after upper limb adaptation to a visuomotor perturbation. We also show that patients with clinically overt motor symptoms do display deficits in the primary adaptation task and these deficits correlate with patients’ clinical symptoms. While the correlation between disease severity and adaptation could provide a clinically meaningful measure of disease progression, the de-adaptation performance could be used as a sensitive early biomarker of SCA6. Future studies will be necessary to expand the present findings to longitudinal analysis and also investigate whether deficits in de-adaptation are a specific feature of pre-clinical SCA6 or apply to other spinocerebellar ataxias.

## Methods

### Subjects

Twenty-five individuals who were heterozygous for the pathogenic expansion in the *CACNA1A* gene were recruited for the present study. All patients were aware of their genetic status. Two patients with a diagnosis of SCA6 were excluded because of difficulty performing the joystick task; data from the remaining twenty-three participants are reported in the present study. Gene positive individuals were sub-divided using the International Co-operative Ataxia Rating Scale (ICARS) kinetic sub-score, which assesses upper and lower limb functions. The pre-clinical group (PRE) was composed of five individuals (Table 1) who had not noticed any symptoms and who had negligible symptoms (Total ICARS ≤ 1). The clinical group (CLIN) was composed of eighteen patients who presented clear ataxic symptoms (Total ICARS > 1, Table 1).

Twenty-one healthy control participants with no history of neurological disease were recruited for this study, although two participants were excluded because of difficulty performing the joystick task. The data from the remaining nineteen control participants are reported. The control participants were divided into two sub-groups, to match in age and number the two groups of SCA6 participants. The control group for the CLIN group is composed of 15 participants (mean age: 64 ± 6.3 years old, seven females, one left-handed participant) and the control group of the PRE group is composed of 5 participants including one who is also in the other control group (mean age: 43.6 ± 15.3 years old, four females, all right-handed). All participants had normal or corrected-to-normal vision. All participants gave their written informed consent prior to the study. Experimental procedures conformed to the Code of Ethics of the World Medical Association (Declaration of Helsinki) and were approved by the National Research Ethics Service (NRES) Committee South Central – Oxford C. Experimental methods were carried out in accordance with the relevant guidelines and regulations.

### Experimental Set-up

Participants sat in an armless chair about 70 cm from a computer screen (size: 26.5 × 16.5 cm) placed vertically in front of them. Presentation of visual stimuli and recordings of cursor movements were performed using PsychoPy^50^. Participants controlled a cursor with a joystick using their dominant hand, the joystick being fixed to a table at a comfortable height on the side of their dominant hand. The joystick was 6.5 cm in height, with a spherical handle of 2 cm in diameter and the maximal centre-out excursion was 17° (RS Components; low profile contactless joystick, APEM 9000 Series). Subjects controlled the joystick using movements of their fingers and/or wrist, and a shield was used to prevent the participants from observing their hand and the joystick while performing the task. The joystick movements controlled a green cursor (diameter: 3 mm) on the computer screen, the position of which was sampled and displayed at 60 Hz. Participants were asked to move the cursor using rapid ballistic movements from the centre of the screen to reach a red target (3 mm) that was presented in a random order to one of eight equidistant positions, separated by 45°, located on the perimeter of a visible circle (radius: 4.6 cm, corresponding to a 3.7° visual angle for the participants – Fig. 1). Subjects were instructed to react quickly to move the cursor to the target, but not stop at the target and rather shoot through it. The red target was presented for 900 ms in its peripheral position, before returning to the centre for another 900 ms. After each trial, participants allowed the cursor to return to its starting position at the centre of the screen by releasing the spring-loaded joystick.

### Experimental design

All groups of participants carried out the same experimental protocol consisting of 4 phases, preceded by a minimum of 20 training trials to familiarise themselves with the task and the use of the joystick. In the first phase (Fig. 1a – Baseline), participants performed 50 baseline trials in which the movements of the joystick and of the cursor matched (no visual perturbation). In the second phase (Fig. 1b – VM Adapt1), the movement of the cursor was rotated anti-clockwise by 60° relative to the joystick movement for 150 trials. The third phase (Fig. 1b – VM Adapt2) was identical to VM Adapt1 but started after a 50-minute rest interval. The last phase (Fig. 1a – De-Adapt) immediately followed VM Adapt2; in this phase the movement of the joystick once again matched the movement of the cursor (i.e. zero rotation) for 150 trials. Thus, acquisition of adaptation was assessed twice: once during VM Adapt 1 and, after a short-term resting break, during VM Adapt 2. The De-Adapt phase allowed assessment of the after-effects of the adaptation. Before the start of VM Adapt 1 and De-Adapt, participants were told about the respective presence and removal of the visual perturbation, but not its exact nature. They were also instructed and reminded for the different blocks to keep making quick ballistic movements toward the target.

### Data analysis

Cursor movements were analysed on a trial-by-trial basis using in-house software written in Matlab (Mathworks Inc, Natick, USA). All kinematic data were filtered with a zero-phase filter with a 25 Hz cut-off and numerically differentiated to determine velocity. The onset and offset of each movement was determined as the point where the velocity crossed 7% of maximum velocity. Reaction time corresponded to the time between target presentation and movement onset. Movement time was calculated as the difference between movement initiation and termination. Movement velocity corresponded to the peak velocity of the movement toward the target. Movement error was quantified as the angle between the line from the starting position to the target and the line from the starting position to the cursor position at peak velocity. Negative values indicate anti-clockwise errors while positive values represent clockwise errors. Mean within-subject values for the different kinematic parameters (reaction time, movement time, movement velocity and error) were created by averaging data over blocks of 10 consecutive trials for each participant. Within-subject movement variability was determined as the standard deviation from the mean error for each block.

### Statistical analysis

Statistical analyses were performed with the SPSS Statistics software package (IBM, Armonk NY, USA). The age, the total ataxic symptoms (ICARS total), the gait and postural symptoms and the kinetic symptoms (ICARS kinetic sub-score) were compared between the two SCA6 individuals groups using Bootstrap for independent samples t-test (1000 sampling). All subsequent analyses were performed separately for the CLIN patients and the PRE individuals, in order to compare their performance to their respective age-matched control group. Movement parameters in baseline (reaction time, movement variability, movement time, movement velocity and movement error) were analysed with two-way ANOVAs with the between-subject factor Group (Control vs SCA6 individuals) and the within-subject factor Block (1 to 5). The average movement errors of every block of 10 trials in VM Adapt1, VM Adapt2 and De-Adapt were analysed with separate two-way ANOVAs with the between-subject factors Group (Control vs SCA6 individuals) and within-subject factor Blocks (1 to 15). To characterise the differences in the adaptation and de-adaptation phase between control and patients, we calculated an Adaptation Index (AI) and a De-Adaptation Index (DeAI) for each participant by averaging the error respectively of the last 30 trials of VM Adapt1 and of the first 30 trials of the de-adaptation phase. These AI and DeAI were compared across SCA6 individuals and their age-matched control group using Bootstrap independent samples t-tests (1000 sampling). For all ANOVAs, Greenhouse–Geisser corrections to the degrees of freedom were applied if Mauchly’s sphericity test revealed a violation of the assumption of sphericity for any of the factors in the ANOVAs. Significant main effects or interaction in the ANOVAs were followed by bootstrapping for independent samples t-test (1000 sampling). To estimate whether there was a link between SCA6 symptoms and adaptation parameters, we correlated the amount of visuomotor adaptation with the total ICARS score as well as with the ICARS kinetic sub-score of the CLIN patients using one-tailed Pearson correlation. The amount of visuomotor adaptation (VM Adapt1) was calculated as follow:

$$VM\,Adapt1=\frac{Error\,First\,block\,-\,Error\,Last\,Block}{Erro\,First\,Block}$$

A similar correlation was ran between DeAI and ICARS total score.

Significance level was set at P < 0.05.
