# Author Correction: Sensorimotor adaptation as a behavioural biomarker of early spinocerebellar ataxia type 6

**DOI:** 10.1038/s41598-018-25324-9

**Published:** 2018-04-30

**Authors:** Muriel T. N. Panouillères, Raed A. Joundi, Sonia Benitez-Rivero, Binith Cheeran, Christopher R. Butler, Andrea H. Németh, R. Chris Miall, Ned Jenkinson

**Affiliations:** 1 Nuffield Department of Clinical Neurosciences, University of Oxford, Oxford, United Kingdom; 2 Department of Experimental Psychology, University of Oxford, Oxford, United Kingdom; 3 Department of Physiology, Anatomy and Genetics, University of Oxford, Oxford, United Kingdom; 4 Department of Clinical Genetics, Churchill Hospital, Oxford University Hospitals NHS Trust, Oxford, United Kingdom; 5 School of Psychology, University of Birmingham, Birmingham, United Kingdom; 6 School of Sport, Exercise and Rehabilitation Sciences, University of Birmingham, Birmingham, United Kingdom

Correction to: *Scientific Reports* 10.1038/s41598-017-02469-7, published online 24 May 2017

The original version of this Article contained a typographical error in the spelling of the author Sonia Benitez-Rivero, which was incorrectly given as Sonia Benitez-Riveiro.

In addition, an incorrect version of the drawing of the joystick in Figure 1 was inadvertently used.

These errors have now been corrected in the HTML and PDF versions of the Article.

